# Lead bioaccumulation in human breast cancer tissue is associated with DNA instability and cell death resistance

**DOI:** 10.1038/s41420-025-02676-6

**Published:** 2025-08-15

**Authors:** Manuel Scimeca, Erica Giacobbi, Rita Bonfiglio, Renata Sisto, Stefano Casciardi, Francesca Servadei, Daniel R. S. Middleton, Julia Bischof, Maria Paola Scioli, Giorgio Modesti, Eleonora Candi, Gerry Melino, Alessandro Mauriello

**Affiliations:** 1 Department of Experimental Medicine, TOR, University of Rome “Tor Vergata”, Rome, Italy; 2 Department of Occupational and Environmental Medicine, Epidemiology and Hygiene, INAIL Research, Monte Porzio Catone, Rome, Italy; 3 Cancer Epidemiology Research Group, Centre for Public Health, Queen’s University Belfast, Belfast, UK; 4 Indivumed GmbH, Falkenried, Hamburg, Germany

**Keywords:** Breast cancer, Breast cancer

## Abstract

Lead (Pb) is increasingly recognized for its potential to alter cellular processes and contribute to cancer development. Although Pb is classified as a probable carcinogen by the IARC, the clinical evidence for its role in breast cancer is inconsistent and limited to epidemiological studies yet. The aim of this study was to investigate the Pb bioaccumulation in human breast cancer tissues by correlating its concentration with specific cancer factors related to carcinogenesis. Biopsy samples from 26 breast cancer patients were collected for molecular investigations (DNA and RNA sequencing), histological analysis, and the assessment of Pb concentration by ICP-MS. Data reported here revealed Pb bioaccumulation in all breast cancer samples, with a significant positive correlation between Pb levels and both Tumoral Mutational Burden and Microsatellite Instability, suggesting an association of Pb with genomic instability in human breast cancer samples. Additionally, Pb was associated with increased expression of cell death-related molecules such as BCL2 and p53. This association allows us to hypothesize a potential involvement of Pb in affecting cell death resistance. Interestingly, Pb concentration showed no correlation to other established prognostic and predictive biomarkers of breast cancer, such as PAM50. Thus, Pb concentration may represent a new independent risk factor for breast cancer development. This study provides new insights into the role of Pb bioaccumulation in breast cancer and suggests that environmental exposure to Pb may contribute to more aggressive tumor behavior through mechanisms involving genomic instability and resistance to apoptosis.

## Introduction

Environmental pollution is a major concern for human health, with heavy metals, such as lead (Pb), posing a significant public health risk worldwide [1–3]. Metals have been extensively studied due to their ability to dysregulate cellular processes through several mechanisms [4–8], which may ultimately contribute to the onset and progression of cancers [9, 10].

The International Agency for Research on Cancer (IARC) evaluated inorganic Pb compounds as ‘possibly carcinogenic to humans’ (Group 2A) based on limited evidence in humans, sufficient evidence in experimental animals [11]. This classification highlights the potential dangers of chronic Pb exposure and its implications for human health. The World Health Organization (WHO) also recognizes the severe health risks posed by Pb exposure. In a 2021 update, the WHO estimated that nearly half of the 2 million deaths attributed to known chemical exposures in 2019 were related to Pb [12]. Nevertheless, the results from human studies still appear to be inconsistent.

Breast cancer has the highest incidence rate of any cancer worldwide, with almost 2.3 million new cases registered in 2022, and the fourth highest number (666,103) of cancer-related deaths [13]. Established risk factors for breast cancer include female sex, age, predisposing genetic mutations (e.g., *BRCA* and *BRCA2*), alcoholic beverages, and hormonal factors that expose breast tissue to higher levels of estrogen, such as oral contraceptives and early menarche, and lack of breastfeeding [14]. Beyond these factors, emerging evidence highlights the role of environmental pollution, including Pb, in breast cancer development [15–18]. In this context, recent evidence shows elevated levels of Pb in the urine and blood of breast cancer patients [19, 20], even if the potential mechanisms through which Pb may contribute to breast carcinogenesis are not yes well established. However, in vitro studies suggest that Pb may lead to cellular damage and alterations in gene expression, also interfering with DNA repair mechanisms [21]. In brief, there is a dearth of robust data on the presence of Pb within human breast cancer tissues and its correlation with specific molecular alterations.

One source of such data is the wealth of archived breast cancer tissue available from histopathology resources. While the source and route of Pb exposure and its position in the causal chain of breast carcinogenesis are often not ascertainable for most biospecimen banks, evaluating Pb bioaccumulation in tumor tissues remains a valuable endeavor. Even in the absence of exposure histories, the characterization of metal burden within cancerous tissues may uncover biologically meaningful patterns that may reflect distinct tumor phenotypes or molecular subtypes, whether because of aetiologic processes or downstream metabolic reprogramming. Moreover, identifying associations between Pb levels and tumor molecular features may have utility in stratifying patients for prognosis, monitoring, or therapeutic decision-making. Thus, the analysis of metal accumulation, even when temporality is uncertain, can provide important clues into tumor biology and may inform both mechanistic hypotheses and clinical management.

With this in mind, we investigated the association between Pb concentrations in human breast cancer tissues and specific carcinogenic hallmark endpoints. Understanding the relationship between Pb and specific molecular features of breast cancer could provide critical insights into its role as a possible risk or prognostic factor, which may have significant implications for preventative, diagnostic, and therapeutic strategies.

## Results

**Fig. 1:**
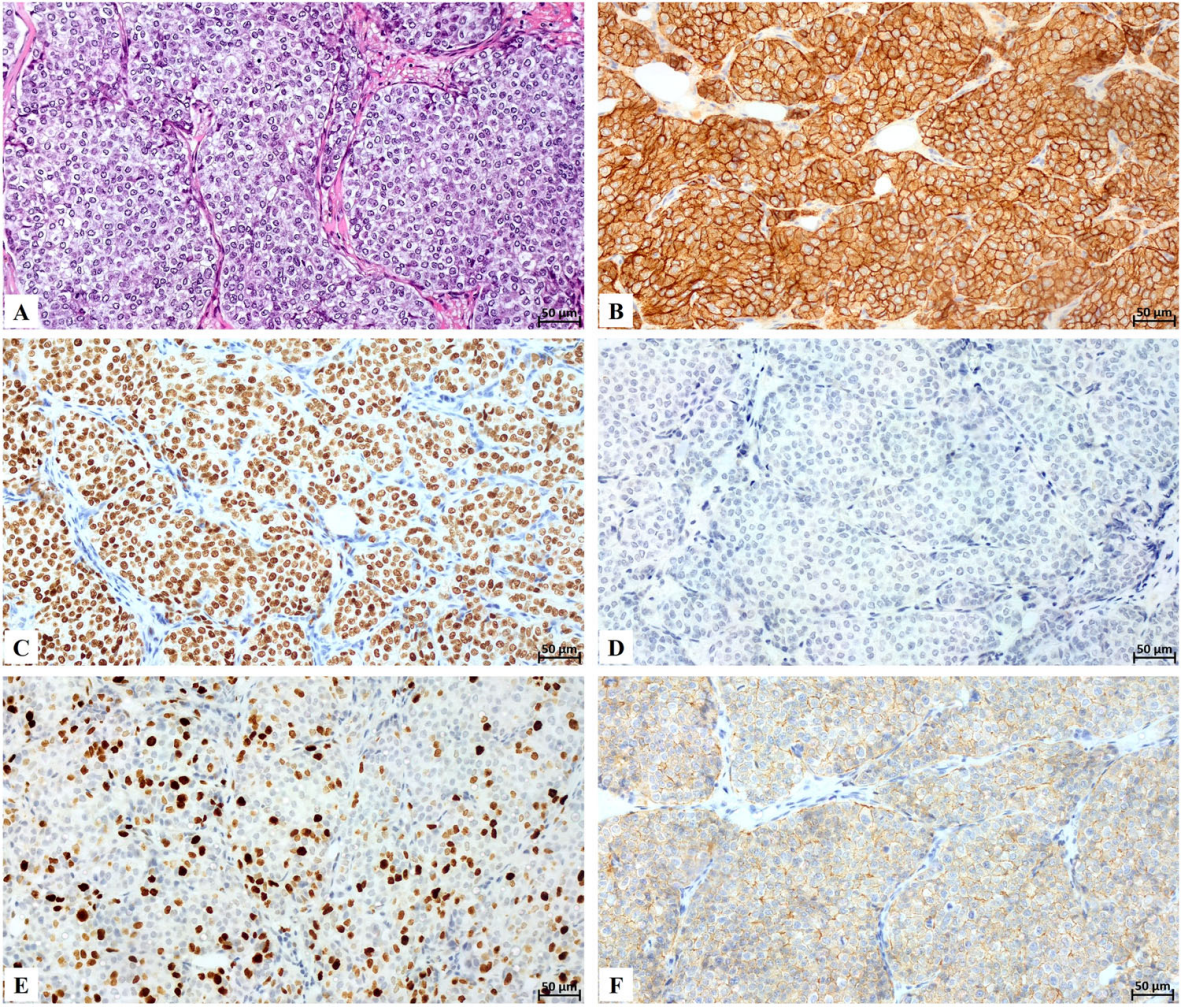
Histological and immunohistochemical analysis of a luminal B breast cancer with high lead (Pb) bioaccumulation. **A** Hematoxylin and eosin staining shows a high-grade infiltrating breast cancer lesion. **B** All breast cancer cells are E-cadherin positive. **C** Estrogen receptor immunostaining displays nuclear positivity in more than 80% of breast cancer cells. **D** The image indicates a lack of positivity for the progesterone receptor. **E** Ki67 receptor immunostaining displays nuclear positivity in more than 20% of breast cancer cells. **F** HER2 status is score 2.

Table 1 describes all patients enrolled in the study with their molecular characterization. Twenty-five cases of invasive ductal carcinoma, one case of in situ ductal carcinoma of the breast (Fig. 1A), of patients aged between 41 and 85 years were examined (mean 59.2 ± 2.9 years). One case was classified as mixed invasive carcinoma with lobular and ductal mixed patterns. According to Nottingham grading [15], we classified the invasive ductal carcinoma as G3 18/25, G2 5/25, and G1 1/25. Concerning the presence of lymph node metastasis, 7 were metastatic, including 3 cases with more than 4 metastatic lymph nodes. Molecular classification allowed us to identify the following subtypes: 9/26 Luminal A, 13/26 Luminal B (Fig. 1B–E), 1/26 normal-like, and 3/26 basal-like.

**Table 1:** Main molecular characteristics of the analyzed breast cancer lesions.

Age	Histological grading	Metastasis	Subtypes PAM50	Microcalcifications	Mutational signatures >0.2
46	3	No	Luminal B	No	Ref Sig MMR1, Ref Sig MMR2
62	3	Sì	Basal-like	No	Ref Sig 3
46	3	Sì	Luminal B (IHC)	yes	Ref Sig 2, Ref Sig 13
43	3	No	Luminal B	No	Ref Sig 2
65	2	No	Luminal A (IHC)	No	-
47	2	No	Luminal A	No	
41	3	No	Luminal B	No	Ref Sig 2
71	3	Sì	Luminal B	No	-
44	2	/	Luminal A	No	
51	3	Sì	Luminal B	No	-
85	3	/	Luminal A (IHC)	No	-
72	2	No	Luminal A	yes	
67	2	No	Luminal A	No	-
41	3	No	Luminal B	No	-
80	3	No	Basal-like	yes	Ref Sig 3
45	2	No	Normal-like	no	Ref Sig 3
78	In situ	No	Luminal A	yes	
51	3	No	Luminal B	yes	Ref Sig 18
44	3	Sì	Luminal B	yes	-
66	3	No	Luminal B	No	-
87	3	/	Luminal B	yes	-
71	2	No	Luminal A (IHC)	no	Ref Sig MMR2
65	3	No	Luminal B	no	-
81	3	/	Basal-like	yes	
44	3	Sì	Luminal A	yes	-
63	1	Sì	Luminal B	No	-

### Determination of Pb concentration in FFPE breast cancers

Quantitative ICP-MS analysis of Pb bioaccumulation detected 100% positivity in investigated cases. Breast cancer FFPE samples showed mean concentrations of 18,2 ± 16,2 mg/kg. A large range of concentrations was found, from a minimum of 0.2 mg/kg to a maximum of 310 mg/kg.

Spearman analysis showed no association between Pb concentration and age (ρs = −0.31; *p* = 0.12).

### DNA instability, cell death, and Pb accumulation

**Fig. 2:**
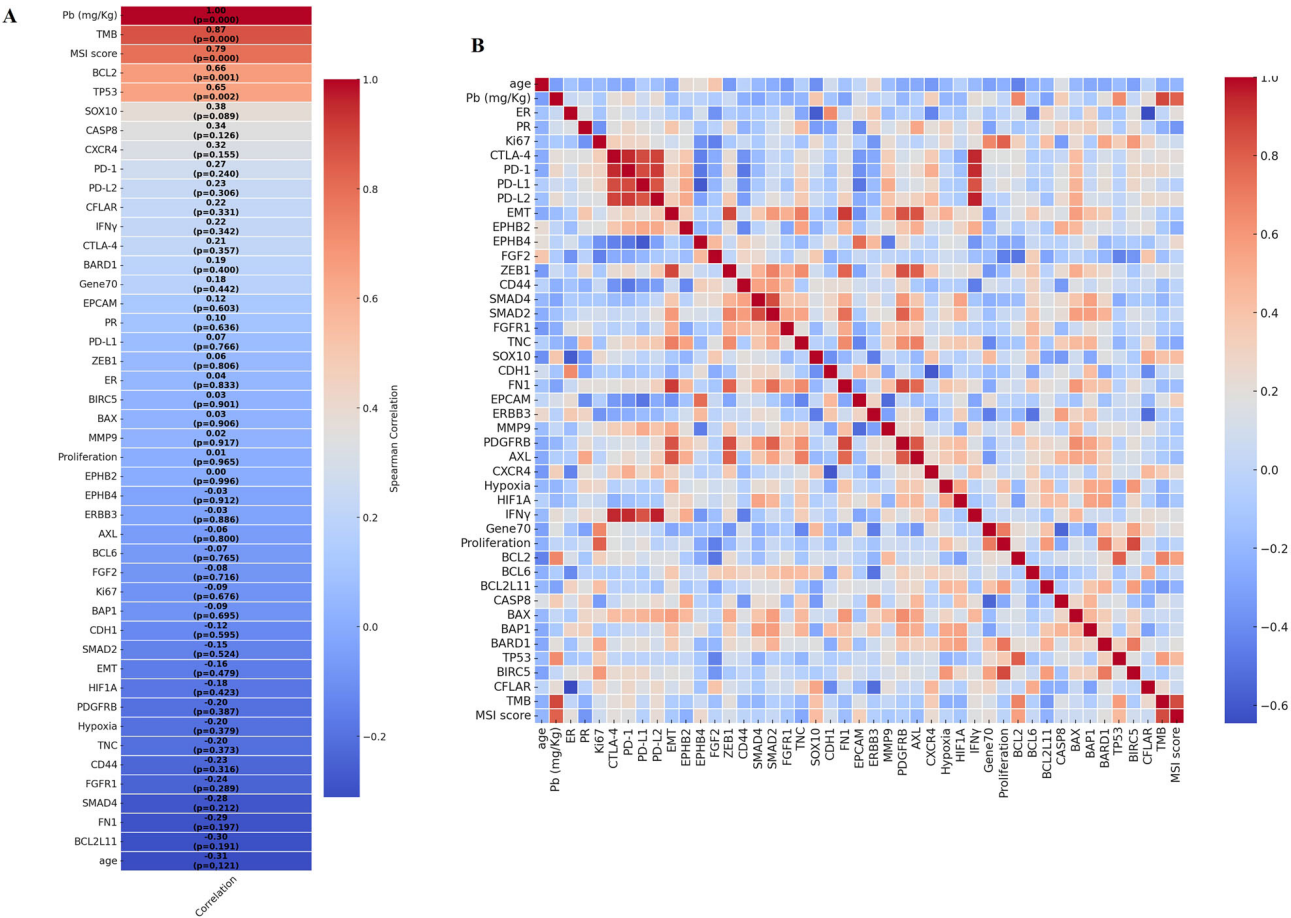
Spearman correlation analysis among continuous variables. **A** The heatmap illustrates the Spearman association between lead concentration (Pb) and the investigated continuous variables. The heatmap reports the ρs and the *p*-value for each association. **B** The heatmap presents the associations among all other investigated variables.

The possible mutational effect of Pb bioaccumulation was evaluated by correlating its concentration with the TMB of breast cancer lesions (Fig. 2A). Spearman analysis showed no association between Pb concentration and age (ρs = −0.31; *p* = 0.12). Noteworthy, a significant positive correlation was found between Pb concentration and TMB (ρs = 0.87; *p* < 0.0001; 95% CI: 0.62–0.98). Similarly, a strong positive association was observed between Pb and MSI score (ρs = 0.79; *p* < 0.0001; 95% CI: 0.49–0.95) (Fig. 2A). Pb concentration also correlated with increased expression of the anti-apoptotic molecule BCL2 (ρs = 0.66; *p* = 0.001; 95% CI: 0.29–0.88) and p53 (ρs = 0.65; *p* = 0.002; 95% CI: 0.23–0.90). These bootstrap-derived intervals support the robustness of these associations despite the sample size. A complete table reporting these correlation coefficients, *p*-values, and bootstrap-derived 95% confidence intervals has been provided as Supplementary Table 1. High p53 expression in samples with Pb could reflect TP53 gene mutations. In fact, our mutational analysis revealed TP53 mutations (Table 1), including both missense mutations and frameshift deletions, in the samples with the highest levels of Pb. The correlation matrix in Fig. 2B shows the association among all other investigated variables.

**Fig. 3:**
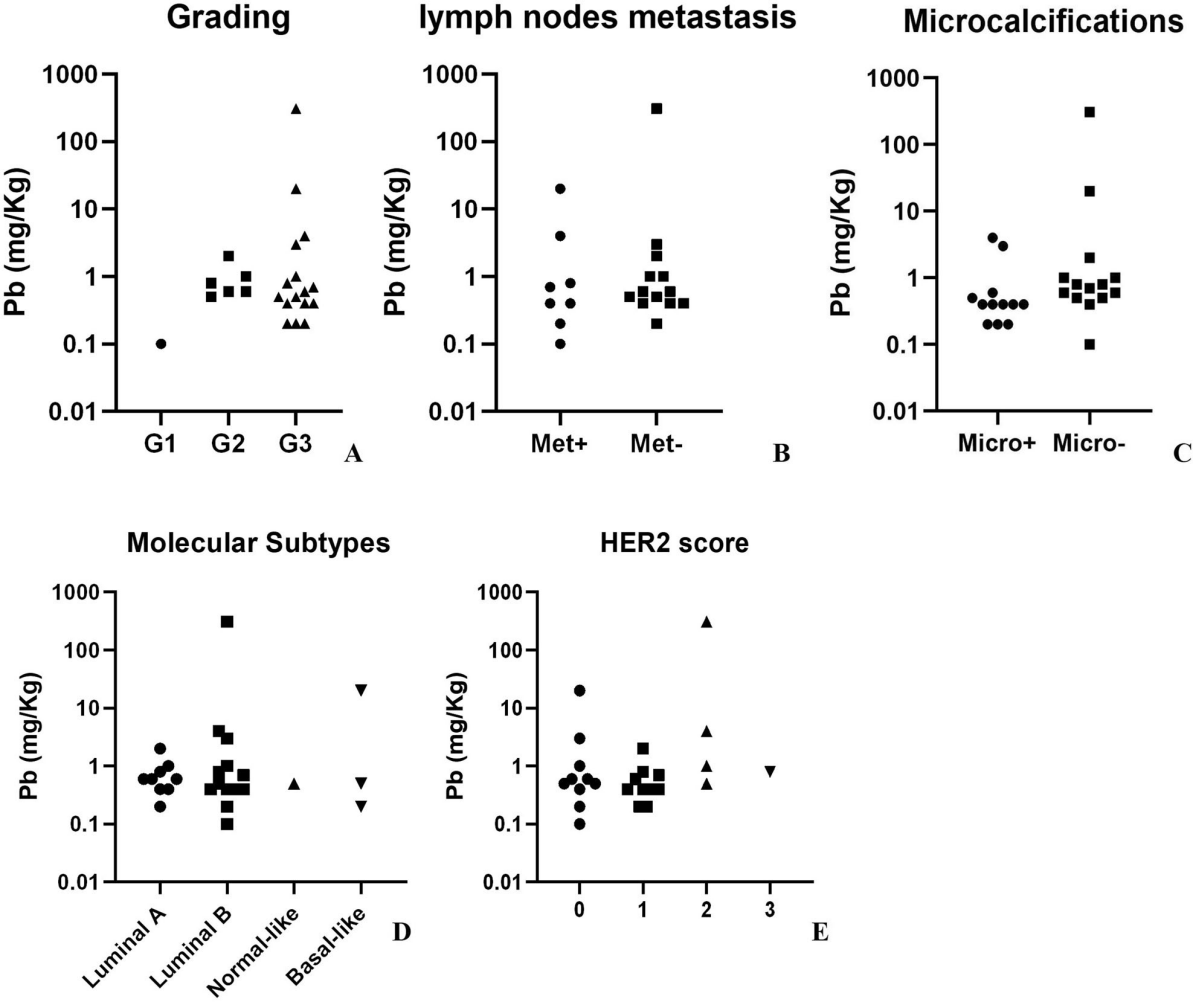
Correlation analysis between lead concentration (Pb) and categorical variables. **A** The graph shows Pb levels in G1, G2, and G3 breast cancer lesions. **B** The graph displays Pb levels in breast cancer lesions of patients with or without lymph node metastasis. **C** The graph illustrates Pb levels in breast cancer lesions with or without microcalcifications. **D** The graph presents Pb levels across different breast cancer molecular subtypes. **E** The graph shows Pb levels according to HER2 scores.

We also studied the possible association between Pb concentration and both clinical and molecular prognostic and predictive biomarkers (Fig. 3). Concerning categorical variables of histological grading (Kruskal–Wallis test *p* = 0.8), the presence of lymph nodes metastasis (Mann–Whitney *U*-Tests *p* = 0.6), microcalcifications (Mann–Whitney *U*-Tests *p* = 0.4), molecular subtypes (Kruskal–Wallis test *p* = 0.99) and HER2 score (Kruskal–Wallis test *p* = 0.2), no significant differences in Pb concentrations were found between categories.

## Discussion

Here we report, to our knowledge, the first evidence of the association between Pb bioaccumulation and carcinogenic mechanistic endpoints in human breast cancer. Molecular investigations revealed a strong positive association between Pb concentration and the genomic instability of breast cancer cells, evaluated in terms of both MSI and TMB. Additionally, Pb concentration was related to a higher expression of molecules involved in cell death mechanisms, such as BCL2 and p53. The increased expression of these molecules could be associated with Pb-related cell death resistance. It is important to underline that Pb concentration was not related to other investigated breast cancer molecular features, including the PAM50 classification. This molecular classification is one of the main parameters currently used for establishing the prognosis and best therapeutic approach for breast cancer patients. The lack of association between Pb accumulation and PAM50 subtypes suggests that Pb may define a distinct biological phenotype. Lead bioaccumulation may reflect environmental or metabolic alterations, such as impaired metal transport, detoxification, or vascularization, that are not specifically associated with known intrinsic genetic subtypes. This observation supports the hypothesis that environmental exposures may contribute to tumor heterogeneity in a manner independent of canonical molecular drivers. It is important to note that, due to the cross-sectional design of this study, causal inferences cannot be made regarding the relationship between Pb bioaccumulation in breast cancer tissue and breast cancer risk. Furthermore, the source of Pb in the tissue cannot be definitively established due to a lack of exposure history among cases. Notably, the absence of correlation between Pb concentration and age suggests that accumulation is not simply a result of lifelong exposure. This raises the possibility that tumor-intrinsic factors, such as deregulated metal uptake or retention mechanisms, may drive Pb bioaccumulation. Potential explanations include increased direct exposure to Pb-containing environmental matrices, lifestyle factors such as cigarette smoking, or reverse causality, whereby Pb accumulation results from dysregulated metal transport mechanisms in cancerous tissue. Previous studies [22] have reported pathological accumulation of Pb and other transition metals in breast cancer tissues compared to healthy controls, suggesting that such accumulation may be linked to the malignant transformation process.

Notwithstanding this caveat, Pb is a widely used heavy metal and legacy pollutant that poses significant health risks to humans; thus, its presence in both cancerous and healthy tissues is warranted. It is commonly found in contaminated air, water, soil, and even in consumer products, largely due to industrial activities, Pb-based paints, and the use of leaded gasoline in the past [23]. This metal is known to be introduced into the body through ingestion or inhalation and absorbed in the gastrointestinal tract. Once absorbed, it can enter the bloodstream and spread to various organs [24]. Based on substantial evidence from in vitro and in vivo studies, which have demonstrated carcinogenic effects at the cellular and organism levels, Pb is currently classified as probably carcinogenic to humans (Group 2A) [11]. Experimental investigations demonstrated its ability to induce DNA breaks and mutations by both direct and indirect mechanisms in several biological contexts (e.g., lung cancer cells, CHO, lymphocytes, mice, rats, and rabbits) but not in breast cancer [25]. The indirect mechanisms are linked to the impairment of the DNA mismatch repair (MMR) system [26]. When the MMR system is inhibited, the cell enters a state of genetic hypermutability known as MSI [27]. Therefore, the observed positive association between Pb levels and both TMB and MSI supports the hypothesis that, in breast cancer, Pb accumulation induces genome instability indirectly by modulating the MMR system, which has obvious implications for cancer progression and potentially prognosis. Indeed, TMB and MSI are currently considered prognostic and predictive biomarkers in several cancers. The use of TMB and MSI as a prognostic marker is justified by the evidence that a higher mutation load may be related to the production of neoantigens, potentially making the tumor recognizable by the immune system [28]. This can increase the likelihood of an effective anti-cancer immune response. It is important to note that the reported frequency of MSI dysregulation in breast cancer is very low [29]. Nevertheless, although breast cancer is generally considered to be less immunogenic compared to other cancers, a possible role of MSI and TMB in predicting response to therapies based on immune-checkpoint inhibitors has been proposed in specific subtypes [29]. The bioaccumulation of Pb in neoplastic tissue appears to identify a small subgroup of breast cancer patients with alterations in TMB and MSI, who may benefit from emerging therapies that target immune system modulation.

Our data also demonstrate that Pb concentration positively correlates with increased expression of the proto-oncogene BCL2 gene and tumor suppressor TP53, whose involvement in the pathogenesis of breast cancer is well established [30]. While TP53 mutations are a well-established mechanism of apoptotic resistance, our finding of a positive correlation between Pb and BCL2 expression suggests that Pb may act via multiple pathways to promote cell survival. It is plausible that Pb might alter the apoptotic threshold by acting on both intrinsic (mitochondrial) and extrinsic apoptotic regulators, facilitating tumor progression through redundancy in anti-apoptotic signaling. BCL2 is known for its role in regulating apoptosis, where its overexpression typically leads to enhanced cell survival and resistance to cell death [30]. On the other hand, TP53, often referred to as the “guardian of the genome,” plays a pivotal role in controlling cell cycle and apoptosis [30–33]. Mutations in TP53 are common in various neoplasia, including breast cancer [30], and are frequently associated with a failure to induce apoptosis, allowing for the unchecked proliferation of damaged cells. The interaction between p53 and BCL2 is a critical component of the apoptotic pathway [34]. p53 can regulate the expression of BCL2 and other pro- and anti-apoptotic proteins, thereby influencing the balance between cell survival and cell death. Our findings suggest that Pb bioaccumulation may dysregulate this delicate balance, potentially through modulating p53 activity and enhancing BCL2 expression. This could lead to a tumorigenic environment where the normal apoptotic response is impaired, allowing cancer cells to evade programmed cell death and continue proliferating.

Given these considerations, it could explain the role of Pb in anti-apoptotic mechanisms underlying the onset and progression of breast cancer, thus having a potential prognostic impact. Specifically, the enhanced expression of BCL2 in the context of high Pb levels could indicate a shift towards anti-apoptotic signaling, which not only supports tumor survival but also confers possible resistance to therapies that rely on inducing apoptosis in cancer cells.

The observed negative correlation between Pb accumulation and the hypoxia score is a paradoxical finding with possible significant implications. Indeed, it is known that heavy metals are generally involved in the generation of ROS and activation of the hypoxia signaling pathway [35]. A possible explanation for this inverse relationship could be that Pb tends to accumulate in more vascularized breast lesions, where the increased blood supply facilitates its deposition. This enhanced vascularization may improve oxygen delivery to the tumor tissue, thereby reducing hypoxic conditions. This could potentially affect the tumor’s aggressiveness, response to treatment, and overall prognosis. However, the exact mechanisms underlying this relationship remain unclear and warrant further investigation.

Overall, the revealed association between Pb concentration and both genomic instability and resistance to cell death is suggestive that elevated Pb levels may indicate a more aggressive tumor. A possible limitation of our investigation is that Pb in FFPE blocks could be introduced during the sample preparation procedures. However, the application of ICP-MS on FFPE tissues has been recently validated in the study of Coyte et al. [36]. By comparing the ICP-MS results from FFPE and fresh tissues, the authors demonstrated that the histological preparation of tissues (formalin fixation, dehydration, and paraffin embedding) does not significantly alter the concentration of numerous bioaccumulated metals, including Pb. A further limitation, as noted, is the study’s cross-sectional design. While not invalidating our results, this design limitation warrants further investigations on tissue samples for which corresponding exposure histories are available, to better establish the source and temporality of Pb exposure, including in comparison to non-cancerous tissue from the same individuals, and from cancer-free controls.

## Conlusions

Environmental pollution is one of the most challenging sustainable development agendas today [1, 37]. Toxic metals are some of the most harmful pollutants to human health [38]. In this challenging context, this study provides novel insights into the potential role of Pb bioaccumulation in breast cancer, revealing significant associations between Pb levels and key molecular features of the disease. Specifically, our findings demonstrate that Pb accumulation is strongly correlated with increased genomic instability, as indicated by elevated TMB and MSI, and cell death resistance due to its positive association with the expression of BCL2 and TP53. This evidence underscores a potential link between Pb exposure and a more aggressive tumor phenotype. Notably, Pb bioaccumulation was found to be independent of other established molecular features, such as the PAM50 classification, suggesting that Pb bioaccumulation could be considered a distinct and independent risk factor in breast cancer patients. The identification of Pb as a potential risk factor highlights the need for increased awareness of environmental exposures in cancer etiology and the importance of monitoring Pb levels in patients with breast cancer.

## Methods

### Samples collection

Breast cancer samples (*n* = 26) were collected from white Italian female patients who underwent open surgery for the presence of breast cancer lesions. Samples were used for molecular (*n* = 21) and histological investigations (*n* = 26). Specifically, a small portion of the tumor (150 mg) from each sample was frozen and had its DNA and RNA sequenced. The remaining portion of the tumor was fixed in formalin and embedded in paraffin. Serial sections from paraffin blocks were used for histological classification according to the 5th edition of the WHO classification of breast tumors [12]; the study of the main breast cancer prognostic and predictive biomarkers (ER, PR, KI67 and HER2) by immunohistochemistry; and the analysis of Pb concentration in situ by Inductively Coupled Plasma Mass Spectrometry (ICP-MS). The study protocol was approved by the Institutional Ethical Committee of the “Policlinico Tor Vergata” (reference number: # 96-19). All experimental procedures were conducted in accordance with the Code of Ethics of the World Medical Association, specifically the Declaration of Helsinki. Informed consent was signed by all patients before surgery.

### Molecular investigation

Fresh frozen tissues were used for whole genome sequencing and RNAseq according to Yang et al. [39]. Microsatellite instability (MSI) classification was determined using the MSIseq R package. Tumoral Mutational Burden (TMB) was calculated as the number of non-synonymous mutations of protein-coding genes divided by exome size in Megabases. PAM50 subtyping as well as risk scores were investigated using the genefu R package [39]. According to PAM50 results, breast cancers were classified as Luminal A, Luminal B, HER2 positive, normal-like, and basal-like breast carcinoma. When PAM50 analysis was unclear, molecular classification was performed by using surrogate immunohistochemical data (ER, PR, Ki67, and HER2). Mutational signatures were calculated using the Mutational Patterns R package.

### Histology

Breast cancer samples were fixed in 4% buffered formalin for 24 h at room temperature and subsequently embedded in paraffin. Four-µm serial sections were stained with hematoxylin-eosin for histological classification. Immunohistochemistry was performed to study the prognostic and predictive biomarkers ER, PR, HER2, and Ki67 (with a 20% cutoff). Briefly, sections were stained using the automated Leica Bond IHC platform (Leica Biosystems, Deer Park, IL). After antigen retrieval, 4-μm-thick sections were incubated with the following primary monoclonal antibodies: mouse monoclonal anti-ER (clone 6F11; Leica Biosystems), mouse monoclonal anti-PR (clone 16; Leica Biosystems), mouse monoclonal anti-Ki67 (clone MM1; Leica Biosystems), and mouse monoclonal anti-HER2 (clone CB11; Leica Biosystems). Reactions were revealed using the BOND-PRIME Polymer DAB Detection System (Leica Biosystems, Deer Park, IL). Immunohistochemistry was evaluated independently by two pathologists who were blinded to one another’s evaluations. For the evaluation of HER2, FISH analysis was performed on breast cancers that were scored as HER2 2+. HER2 amplification was established according to ASCO/CAP guidelines [40].

### Pb determination by ICP-MS

ICP-MS analysis was performed by Agri-Bio-Eco Laboratori Riuniti S.R.L. Briefly, four sections of 20 µm thickness were obtained from each FFPE sample. Sections were stored in 1.5 mL Eppendorf tubes, and xylene was added and left overnight to allow paraffin digestion. The xylene was then replaced twice, followed by three rinse cycles of ultra-pure ethanol to completely remove paraffin residues. Thereafter, complete evaporation of ethanol was performed to fully dry the samples. The appropriately weighed samples were digested in a 1:10 solution composed of hydrogen peroxide (H_2_O_2_) and nitric acid (HNO_3_). The digested sample was made up to the mark of 10 mL and analyzed by the ICP-MS (Agilent 7700 series) technique.

### Statistical analysis

The Shapiro–Wilk test revealed that Pb did not follow a normal distribution. Consequently, Spearman’s rank correlation coefficients were calculated to evaluate the associations between Pb concentrations and the selected continuous variables. To further address concerns about the small sample size, 95% confidence intervals (CIs) for the Spearman ρ-values were calculated using a bootstrap approach (1000 iterations). This allowed for a more robust assessment of the variability of the correlation estimates. Bonferroni correction was applied to adjust for multiple testing, setting the significance threshold at *p* < 0.05. Correlation coefficients (ρ), *p*-values, and the bootstrap-derived 95% confidence intervals were reported for each pairwise comparison. To illustrate the strength and direction of associations, a heatmap was generated with color gradients representing the magnitude and direction of the associations. Positive correlations are indicated in shades of red, while negative correlations are shown in shades of blue.

Kruskal–Wallis or Mann–Whitney *U*-Tests were performed to associate Pb concentration with categorical variables lymph nodes metastasis (yes/no), the presence of microcalcifications (yes/no), histological grading (G1, G2, G3), subtypes PAM50 (Lumina A, Luminal B, HER2 positive, normal-like and basal-like) and HER2 score (0–3).

## Supplementary information

Supplementary Table 1 Bonferroni-adjusted *p*-values and 95% confidence intervals for the analysis between Pb and other variables.

## Data Availability

All data are included in the manuscript or available from the corresponding authors upon reasonable request.
