# Supplementary material for: Lead bioaccumulation in human breast cancer tissue is associated with DNA instability and cell death resistance
Source: Cell Death Discov. 2025 Aug 15;11:383. doi: 10.1038/s41420-025-02676-6 (PMC12356885; doi:10.1038/s41420-025-02676-6)
Supplement: Supplementary file 1 — Supplementary Table 1 Bonferroni-adjusted p-values and 95% confidence intervals for the analysis between Pb and other variables. [file 41420_2025_2676_MOESM1_ESM.docx]

Supplementary Table 1 Bonferroni-adjusted p-values and 95% confidence intervals for the analysis between Pb and other variables.

| Variable | Bonferroni-adjusted p-value | 95% CI lower | 95% CI upper |
| --- | --- | --- | --- |
| TMB | 0,00 | 0,62 | 0,99 |
| MSI score | 0,00 | 0,49 | 0,95 |
| BCL2 | 0,05 | 0,30 | 0,88 |
| TP53 | 0,07 | 0,23 | 0,90 |
| SOX10 | 1,00 | -0,08 | 0,75 |
| CASP8 | 1,00 | -0,12 | 0,73 |
| CXCR4 | 1,00 | -0,10 | 0,63 |
| PD-1 | 1,00 | -0,15 | 0,67 |
| PD-L2 | 1,00 | -0,17 | 0,60 |
| CFLAR | 1,00 | -0,21 | 0,58 |
| IFNγ | 1,00 | -0,21 | 0,65 |
| CTLA-4 | 1,00 | -0,24 | 0,61 |
| BARD1 | 1,00 | -0,30 | 0,61 |
| Gene70 | 1,00 | -0,27 | 0,59 |
| EPCAM | 1,00 | -0,29 | 0,47 |
| PD-L1 | 1,00 | -0,37 | 0,52 |
| ZEB1 | 1,00 | -0,45 | 0,57 |
| Ki67 | 1,00 | -0,41 | 0,51 |
| BIRC5 | 1,00 | -0,41 | 0,50 |
| BAX | 1,00 | -0,45 | 0,51 |
| MMP9 | 1,00 | -0,40 | 0,46 |
| Proliferation | 1,00 | -0,40 | 0,47 |
| EPHB2 | 1,00 | -0,42 | 0,41 |
| EPHB4 | 1,00 | -0,47 | 0,38 |
| ERBB3 | 1,00 | -0,53 | 0,40 |
| ER | 1,00 | -0,46 | 0,42 |
| PR | 1,00 | -0,50 | 0,40 |
| AXL | 1,00 | -0,56 | 0,47 |
| BCL6 | 1,00 | -0,52 | 0,39 |
| FGF2 | 1,00 | -0,54 | 0,40 |
| BAP1 | 1,00 | -0,52 | 0,36 |
| CDH1 | 1,00 | -0,59 | 0,34 |
| SMAD2 | 1,00 | -0,60 | 0,37 |
| EMT | 1,00 | -0,64 | 0,32 |
| HIF1A | 1,00 | -0,58 | 0,26 |
| age | 1,00 | -0,49 | 0,24 |
| PDGFRB | 1,00 | -0,64 | 0,25 |
| Hypoxia | 1,00 | -0,65 | 0,33 |
| TNC | 1,00 | -0,63 | 0,28 |
| CD44 | 1,00 | -0,60 | 0,22 |
| FGFR1 | 1,00 | -0,63 | 0,22 |
| SMAD4 | 1,00 | -0,71 | 0,21 |
| FN1 | 1,00 | -0,65 | 0,15 |
| BCL2L11 | 1,00 | -0,65 | 0,14 |
